# The Paradox of Mental Health: Over-Treatment and Under-Recognition

**DOI:** 10.1371/journal.pmed.1001456

**Published:** 2013-05-28

**Authors:** The PLOS Medicine Editors

## Abstract

The *PLOS Medicine* editors discuss the paradox of mental health, where over-diagnosis and treatment of some mental health issues exists alongside profound under-recognition of mental health conditions in the developing world.

*Please see later in the article for the Editors' Summary*

Among all the conditions in the world of health, mental health occupies a unique and paradoxical place.

On the one hand is over-treatment and over-medicalization of mental health issues, often fueled by a pharmaceutical industry interested in the broadening of the boundaries of “illness” and in the creation of more and wider diagnostic categories and thus markets for “selling sickness.” On the other hand exists profound under-recognition of the suffering and breadth of mental health issues affecting millions of people across geographies, which is a global problem.

As a journal, *PLOS Medicine* has covered both sides of the mental health “coin,” and we continue to make mental health in general a priority area. We recognize that the whole of the field of mental health research is relatively underdeveloped, and that a particular scarcity of clinical trials exists from outside high-income settings and for non-drug interventions. As a result, we also support efforts to improve capacity in mental health research whilst committing to the publication of the state of the art in research and commentary [1],[2].

Over-treatment, especially when it results from “disease mongering,” is a persistent and troubling issue. The harms of over-treatment arise from situations where normal life experiences (such as menopause, shyness, grief, etc.) are deemed illnesses [3] or when diseases are “created” from mild problems and symptoms (such as restless legs *syndrome* or female sexual *dysfunction*) [4],[5]. In both situations, people become patients, and their problems are deemed to need medical treatment when they may not need it or could be harmed by it, or when nonmedical options are available. Over-diagnosis and over-treatment have been shown for a range of human conditions [3], but this phenomenon as it relates to mental health is particularly powerful [6]. For example, the widespread over-diagnosis of conditions such as bipolar disorder, autism spectrum disorder, and attention deficit hyperactivity disorders (ADHD), especially among children, is now being documented—the US Centers for Disease Control recently estimated that 6.4 million children aged 4 to 17 had received an ADHD diagnosis at some point in their lives (amounting to 11% of all US children)—a 41% increase in the last decade that has been met with alarm and concern by many doctors and parents [7]. Two thirds of these children are said to be on medication for the condition. Recent Canadian data [8] reaffirm the concerns with excessive labeling of normal child behavior as pathological. Over-diagnosis in mental health risks unnecessary tests and treatment, the stigma associated with being labeled mentally ill, and the considerable costs of testing, treatment, and wasting resources that could be better utilized elsewhere [3],[5].

The recent DSM-5 process is a lightning rod for these concerns: this month's update of the psychiatric diagnostic manual has been widely criticized for continuing the tradition of broadening diagnostic categories and adding new conditions that redefine more people as having mental illness and in need of pharmaceutical treatment [9],[10]. That decisions about DSM-5 categories are made by experts with financial ties to the industry that benefits most from a widened patient population [11],[12], is particularly worrying.

In perhaps the most dedicated venue for discussions of this topic, the Selling Sickness conferences (http://www.sellingsickness.com), which *PLOS Medicine* has been instrumental in shaping, have brought together academic researchers, medical reformers, consumer advocates, and health journalists with shared interests in examining the problem of disease mongering and developing strategies and coalitions for change. The inaugural conference in 2006 coincided with our launch of the *PLOS Medicine* Disease Mongering Collection (http://bit.ly/18i6j6h) that to this day remains astonishingly relevant. In February 2013 we participated again, this time in a roundtable on the role of the medical media where we outlined our responsibility as editors to avoid the spin in published articles and the journal's press releases that can fuel hype about new disease categories and treatment [13]; we also highlighted another important role of journals in fighting disease mongering: to require that all clinical trials be registered and data be reported and shared, so that the full picture of the benefits and harms of tested interventions can be seen (see, for example, http://www.alltrials.net). The conference's Call to Action petition (http://sellingsickness.com/final-statement/) is available for readers to view and sign. Later in 2013, two comrade conferences, PharmedOut (http://www.pharmedout.org/) and Avoiding Overdiagnosis (http://www.preventingoverdiagnosis.net/), will continue the conversation about both the extent and the prevention of over-diagnosis, and will undoubtedly provide new insights into the problems associated with over-treatment of mental health.

Equally important, however, is the vast *under*-recognition of mental health conditions, especially in the developing world. This neglect has occurred at multiple levels including at the national level, where many countries have failed to establish adequate mental health policy. At the level of global health agendas, mental health was essentially ignored in the Millennium Development Goal program and failed to elevate to prominence at the recent United Nations special assembly on non-communicable disease.

As many others have noted [14]–[16], this neglect makes little sense: more than 13% of the global burden of disease is attributable to neuropsychiatric disorders, and over 70% of this burden lies in low- and middle-income countries (LMICs). Almost a quarter of the world's disability burden is now attributable to mental and behavioral disorders (including depression, anxiety, Alzheimer disease, and schizophrenia) [17]. And yet mental health has failed thus far to receive the political priority and international funding commensurate with its global toll [14]. There are signs this tide is shifting, and several prominent groups and organizations are working to raise the profile of global mental health. *PLOS Medicine* has provided a forum for that effort over the last few years, publishing packages of care for mental health disorders in LMICs [18] and an ongoing series on mental health interventions in practice [2]. And this week we conclude a five-part series that sets out an agenda for integrating mental health care into primary care, maternal health, non-communicable disease, and HIV interventions in the developing world [19]. All of these analyses were done by researchers free of financial links to manufacturers with a stake in expanded markets, thus providing the necessary independent opinion.

In addition, we've recently published high-quality research on a range of topics within mental health that contributes to improved clinical practice, policy, and action. This includes definitive evidence on the long-term health consequences of sexual abuse [20] and trafficking [21], a genome-wide analysis establishing the limited ability of genetic data to predict antidepressant response [22], and a meta-analysis reporting the relative benefits and harms of adjunctive antipsychotic medications in depression [23]. These studies add to a growing evidence base, and signal a growing recognition of the importance of mental health.

Still, our understanding of all aspects of mental health is relatively underdeveloped. As others have acknowledged [3],[24], the research base for over-diagnosis and harm from over-treatment remains limited, and so the new initiatives and calls for action are welcomed. So too is growing recognition and research on genuine mental health issues and the best ways to address and prevent mental health problems, especially in terms of policy and human rights action and in a global context. To the extent that these two areas (*over-treatment* on one hand, *under-recognition* on the other hand) represent the paradox of mental health, where's the balance point? We don't have all the answers, but as a journal we reaffirm our commitment to publishing rigorous, insightful research and commentary on the breadth of issues around global mental health, and we welcome continued debate on the challenges this paradox represents. The largest challenge may be to recognize and prioritize mental health globally—with the requisite political visibility, funding, research, and attention—without reducing it to an object for disease mongering, pathologizing, and harmful over-treatment.

## Abbreviations

ADHD: attention deficit hyperactivity disorders
LMIC: low- and middle-income country
